# Decorticate, decerebrate and opisthotonic posturing and seizures in Kenyan children with cerebral malaria

**DOI:** 10.1186/1475-2875-4-57

**Published:** 2005-12-07

**Authors:** Richard Idro, Godfrey Otieno, Steven White, Anderson Kahindi, Greg Fegan, Bernhards Ogutu, Sadik Mithwani, Kathryn Maitland, Brian GR Neville, Charles RJC Newton

**Affiliations:** 1 Centre for Geographic Medicine Research-Coast, Kenya Medical Research, Kenya; 2 Department of Paediatrics and Child Health, Mulago Hospital/Makerere University, Kampala, Uganda; 3 Department of Clinical Neurophysiology, Great Ormond Street Hospital for Children London, UK; 4 Infectious Disease Epidemiology Unit, Department of Infectious and Tropical Diseases, London School of Hygiene and Tropical Medicine, London, UK; 5 Department of Paediatrics, Faculty of Medicine and the Wellcome Trust Centre for Tropical Medicine, Imperial College, London, UK; 6 Neurosciences Unit, the Wolfson Centre, Institute of Child Health, University College London, UK

## Abstract

**Background:**

Abnormal motor posturing is often observed in children with cerebral malaria, but the aetiology and pathogenesis is poorly understood. This study examined the risk factors and outcome of posturing in Kenyan children with cerebral malaria.

**Methods:**

Records of children admitted to Kilifi district hospital with cerebral malaria from January, 1999 through December, 2001 were reviewed for posturing occurring on or after admission. The clinical characteristics, features of raised intracranial pressure, number of seizures and biochemical changes in patients that developed posturing was compared to patients who did not.

**Results:**

Of the 417 children with complete records, 163 (39.1%) had posturing: 85 on admission and 78 after admission to hospital. Decorticate posturing occurred in 80, decerebrate in 61 and opisthotonic posturing in 22 patients. Posturing was associated with age ≥ 3 years (48.1 vs 35.8%, *p *= 0.01) and features of raised intracranial pressure on funduscopy (adjusted OR 2.1 95%CI 1.2–3.7, *p *= 0.009) but not other markers of severity of disease. Unlike decorticate posturing, decerebrate (adjusted OR 1.9 95%CI 1.0–3.5) and opisthotonic posturing (adjusted OR 2.9 95%CI 1.0–8.1) were, in addition, independently associated with recurrence of seizures after admission. Opisthotonus was also associated with severe metabolic acidosis (OR 4.2 95%CI 3.2–5.6, *p *< 0.001). Thirty one patients with posturing died. Of these, 19 (61.3%) had features suggestive of transtentorial herniation. Mortality and neurological deficits on discharge were greatest in those developing posturing after admission.

**Conclusion:**

Abnormal motor posturing is a common feature of cerebral malaria in children. It is associated with features of raised intracranial pressure and recurrence of seizures, although intracranial hypertension may be the primary cause.

## Background

Cerebral malaria is one of the most common non-traumatic encephalopathies affecting children worldwide and the most severe neurological complication of falciparum malaria [1,2]. Patients present with a one to three day history of fever, seizures, unarouseable coma and brainstem signs [1,3,4]. Abnormal motor posturing (AMP), manifesting as decorticate, decerebrate or opisthotonus, is common, but the aetiology and pathogenesis of these signs in cerebral malaria and the prognosis of each type of posturing is poorly understood [3-9]. Raised intracranial pressure (ICP), cerebral ischaemia, hypoxia, hypoglycaemia, and hyponatraemia are recognized causes of AMP [10,11] and raised ICP is a feature of cerebral malaria in African children [6,7,12]. In a study of cerebral malaria in which ICP was monitored, all children in whom AMP was documented had raised ICP, but in some patients, the timing of the AMP was not directly associated with episodes of severe intracranial hypertension [7]. Based on current guidelines, children with similar features presenting to emergency services in areas where malaria is absent will receive management for raised ICP, including paralysis and ventilation. On the other hand, seizures have been described in over 60% of patients with cerebral malaria and other acute encephalopathies associated with AMP [10,13]. The hypothesis that AMP may be caused by seizures has led to the use of anticonvulsants in their management but there is little evidence to support this management. Other treatment practices have included non-interventional observation or therapy with mannitol.

The frequent occurrence of AMP in children with cerebral malaria, lack of ventilation facilities in most centres that manage such patients and the apparent resolution in most patients without active intervention warrants further description of the associated risk factors. In this study, records of children admitted to a Kenyan district hospital with cerebral malaria were examined to determine the risk factors associated with AMP, in particular to determine any association between AMP and features of raised ICP or seizures. The relationship between AMP and outcome was also examined.

## Methods

### Study design

Clinical records of all children admitted to Kilifi District Hospital with cerebral malaria from January, 1999 through December, 2001 were examined for AMP. This hospital is situated in a malaria-endemic area and the setting was described previously [14,15].

### Definition of cerebral malaria

Cerebral malaria was defined as unarouseable coma (unable to localize a painful stimulus, Blantyre Coma Score ≤ 2) [3], at least one hour after termination of a seizure, administration of diazepam or correction of hypoglycaemia, with asexual forms of falciparum malaria parasites on a Giemsa stained blood smear and cerebrospinal fluid (CSF) examination not suggestive of bacterial meningitis [2,16]. Children with epilepsy, cerebral palsy or sickle cell disease were excluded.

### Definition of decorticate, decerebrate and opisthotonic posturing

Abnormal motor posturing, classified as decorticate, decerebrate or opisthotonic posturing is characterised by generalised extension of the trunk and lower limbs with increased muscular tone [11]. Decorticate posturing was defined as semi-flexion, adduction and internal rotation at the shoulders and semi-flexion or flexion at the elbows. Decerebrate posturing was defined as extension of upper limbs, adduction and internal rotation of the shoulders, with pronation of the forearms. Opisthotonic posturing was defined as decerebrate posturing where the neck and back are arched posteriorly [10]. For the purposes of the study, if a patient exhibited more than one type of AMP, the more severe type ordered as: decorticate, decerebrate and opisthotonus was assigned.

### Admission procedures

Ethical permission for the study was obtained from the Kenya Medical Research Institute Scientific and Ethical Review Committees. At admission, standard proforma were completed detailing the medical history and physical examination. Emergency care and resuscitation were performed according to standard protocols [2,17]. The number, duration and type of seizures prior to admission were documented. Level of coma was assessed using the Blantyre coma scale (BCS) [3]. Features of AMP were documented and assigned to decorticate, decerebrate or opisthotonic categories.

### Laboratory procedures

Patients had venous blood drawn for a full blood count and blood glucose, parasite density, plasma electrolytes and microbiological culture. A heparinized venous sample was drawn to determine acid base status. Lumbar punctures were performed when the level of consciousness improved (BCS>2) and the child did not have brainstem signs[5].

### Inpatient course

While in the ward, patients were closely monitored during the first hour. Thereafter, clinical assessments, level of consciousness and blood glucose measurements were repeated every four hours by the nursing staff. Physicians assessed the children at four and 24 hours and on a daily basis thereafter. Patients were re-assessed if the nursing team observed seizures, AMP, deterioration in consciousness or worsening vital signs. Seizures on admission and those that developed after admission and lasted longer than five 5 minutes were treated with intravenous diazepam 0.3 mg/kg. Paraldehyde (0.4 ml/kg intramuscularly) was the second line antiepileptic drug, and phenytoin (18 mg/kg intravenously) or phenobarbital (18 mg/kg) were the third line antiepileptic drugs. Clinical signs within one hour of a seizure or anticonvulsant were not reported. Observed seizures were documented, timed and classified as partial, partial with secondary generalization or generalized tonic-clonic and recorded on a proforma sheet. Direct ophthalmoscopy for retinal signs was performed on admission, at 4 hours and then daily until full consciousness was regained by the attending physicians. In the first year, some of these examinations were performed by an ophthalmologist who trained the other physicians in the recognition of abnormal retinal features, but there was no formal validation of the quality of funduscopy performed by the attending physicians against that of the ophthalmologist. AMP and other brainstem signs were documented. Treatment and nursing care were according to standard protocols [2,17]. Patients were deemed to have regained full consciousness when they scored a BCS of 5 (or BCS 4 in children <9 months) [18].

### Cerebral function analyser monitoring

Fifty-eight of the study patients participated in pharmacokinetic studies of anti-epileptic drugs [19,20] and had continuous monitoring with a 4-channel cerebral function analyser (CFAM3c RDM, East Sussex) for the duration of coma. The CFAM recordings were examined for evidence of seizure activity and changes in amplitude during episodes of AMP.

### Data management and statistical analysis

Patients' records were entered directly into a database using FoxPro software and analysed using SPSS 11.5 (SPSS Chicago Inc, 2003). Patients were divided into three groups: those with AMP at admission, those with AMP after admission and those without. Children who had AMP at admission and additional episodes after admission were regarded as having AMP at admission and the most severe form of AMP was assigned to the patient. Univariate analysis was performed to identify features associated with AMP. Patients who had AMP on admission were compared with those without AMP. A similar comparison was also made between patients posturing after admission and those without AMP. Categorical variables were compared using Pearson's Chi square or Fisher's exact test (2-tailed) as appropriate. Continuous variables were compared with the student's t-test. The mean parasite densities were compared after logarithmic transformation and the median and inter quartile range (IQR) used for other skewed data. Logistic regression analysis was performed to identify risk factors independently associated with the development of AMP.

## Results

### General description

A total of 15,506 children were admitted to KDH during the three-year period. Of these, 5,767 had malaria as a primary diagnosis. Four hundred and twenty-six patients fulfilled the criteria for cerebral malaria, of whom nine had incomplete records and were excluded. Of the remaining four hundred and seventeen, 215 (51.6%) were males. The median age was 27 (IQR 15–41) months and median duration of fever prior to admission was two (IQR one – three) days. Overall, 163 children (39.1%) had AMP: 85 (20.4%) presented with AMP and an additional 78 (18.7%) developed AMP after admission. Of the 85 patients who had AMP on admission, 63 (74%) had further episodes after admission. Most AMP occurred spontaneously and manifested as decorticate posturing in 80 (49.1%), decerebrate posturing in 61 (37.4%) and opisthotonus in 22 (13.5%).

### Presentation and risk factors for abnormal motor posturing at admission

Admission clinical characteristics of patients with AMP at admission, during admission or without AMP are summarized in Table 1 (and clinical features of patients with and without AMP are in the attached Additional file 1). Overall, patients with AMP were older: 48.1% of those three years or older had AMP compared to only 35.8% of children under three years, *p *= 0.01. No association was observed between AMP and duration of illness, history of seizures or parasite density. No relationship was also observed between AMP and other markers of severity of illness at admission such as a history of multiple convulsions, profound coma, deep breathing, hypotension, hypoglycaemia, severe anaemia, hyponatraemia or hyperparasitaemia. No significant differences were found in the CSF white blood cell count, protein, sugar or CSF: blood sugar ratio. Using multiple logistic regression analysis, age three years or older was independently associated with a child presenting with AMP (adjusted OR 2.0 95% CI 1.7 – 2.4, *p *< 0.001). Unlike decorticate or decerebrate posturing, opisthotonic posturing was also independently associated with severe acidosis, (adjusted OR 4.2 95% CI 3.2–5.6, *p *< 0.001).

**Table 1:** Characteristics of patients with posturing

**Clinical and laboratory characteristics, and changes during admission**	**Patients with no posturing (n = 254)**	**Patients posturing at admission (n = 85)**	**P value**	**Patients posturing after admission (n = 78)**	**P value**
**Clinical characteristics on admission**					
Sex, male (%)	130 (51.2)	43 (50.6)	0.93	42 (53.8)	0.68
Mean (SD) age in months	28.4(20.1)	38.0(25.7)	0.01	35.0 (21.0)	0.01
Median (IQR) duration of illness in days	2.0 (1 – 3)	2.0 (1 – 3)	-	2.0 (0.3 – 3)	-
History of seizures, (%)	217 (85.4)	72 (85.7)	0.95	66 (84.6)	0.86
Median (IQR) duration of coma before admission in hrs	3 (2 – 6)	3 (2 – 6)	-	4 (2 – 6)	-
Mean (SD) axillary temperature °C	38.0 (4.3)	38.7 (7.0)	0.25	38.8 (7.2)	0.22
Deep (acidotic) breathing, (%)	69 (27.2)	26 (30.6)	0.54	24 (30.8)	0.54
Profound coma (BCS 0, (%))	58 (22.8)	15 (17.6)	0.31	19 (24.4)	0.78
Retinal haemorrhages, (%)	18 (7.1)	15 (17.6)	0.01	11 (14.1)	0.07
**Laboratory investigations on admission**					
Severe anaemia, (haemoglobin < 5.0 g/dl, (%))	54 (22.3)	17 (20.5)	0.73	14 (18.4)	0.47
Hypoglycaemia, (Glucose < 2.2 mmol/L, (%))	51 (21.3)	14 (17.7)	0.50	17 (23.3)	0.71
Severe acidosis, (Base excess < – 15, (%))	56 (24.6)	19 (25.3)	0.89	23 (32.9)	0.17
Hyponatraemia, (Plasma sodium < 135 mmol/L, (%))	114 (47.7)	39 (51.3)	0.58	32 (43.8)	0.56
Mean log10 (SD) of parasite density	4.8 (1.2)	4.8 (1.2)	0.77	4.7 (1.3)	0.39
Hyperparasitaemia, (> 20% parasitaemia, (%))	77 (31.8)	22 (26.5)	0.36	24 (31.6)	0.97
**Brainstem features of raised ICP observed during admission**					
Deterioration in level of consciousness, (%)	64 (25.2)	39 (45.9)	<0.01	43 (55.1)	<0.001
Sluggish pupillary reaction, (%)	82 (32.4)	37 (43.5)	0.06	30 (38.5)	0.32
Non-reactive pupils, (%)	12 (4.7)	3 (3.5)	0.77*	4 (5.1)	1.00*
Pupillary sizes, (%)					
Constricted	42 (16.5)	6 (7.1)	0.03	8 (10.3)	0.18
Dilated	41 (16.2)	24 (28.6)	0.01	19 (24.4)	0.10
Unequal	3 (1.2)	3 (3.5)	0.17*	3 (3.8)	0.14*
Dysconjugate gaze, (%)	42 (17.1)	28 (33.3)	<0.01	20 (26.0)	0.08
Congested retinal veins, blurred disc margins or overt papilloedema, (%)	16 (6.3)	14 (16.5)	<0.01	18 (23.1)	<0.001
Horizontal oculocephalic eye deviation, (%)					
No deviation	17 (6.7)	5 (5.9)	0.79	6 (7.7)	0.76
Minimal deviation	44 (17.3)	16 (18.8)	0.75	11 (14.1)	0.50
Abnormalities in respiration, (%)					
Irregular	49 (19.3)	18 (21.2)	0.71	20 (25.6)	0.23
Shallow	42 (16.5)	6 (7.1)	0.03	14 (17.9)	0.77
Respiratory arrest	12 (4.7)	9 (10.6)	0.05	10 (12.8)	0.01
**Seizures**					
Clinical seizures witnessed in the ward, (%)	29 (50.8)	49 (57.6)	0.27	58 (74.4)	<0.001
Type of witnessed seizures, (%)					
Partial	74 (29.1)	28 (32.9)	0.51	38 (48.7)	<0.01
Partial with secondary generalisation	29 (11.4)	11 (12.9)	0.71	16 (20.5)	0.04
Generalised	81 (31.9)	28 (32.9)	0.86	39 (50.0)	<0.01
**Outcome**					
Mortality, (%)	32 (12.6)	15 (17.6)	0.24	16 (20.5)	0.08
Neurological deficits, (%)	17 (6.7)	7 (8.2)	0.63	15 (19.5)	<0.01

* Fisher's exact test (two tailed)

### Abnormal motor posturing after admission

Of the 78 children who developed posturing after admission, 38 (49%) had decorticate, 30 (38%) decerebrate and 10 (13%) opisthotonic posturing. This distribution was similar to the overall distribution of AMP. Apart from being younger, no differences in admission characteristics and in particular, the duration of illness or coma, history of seizures, level of coma, parasite density, blood glucose, electrolytes, acid base status or haemodynamic state were observed when compared to those who presented with AMP (Table 1).

**Figure 1:**
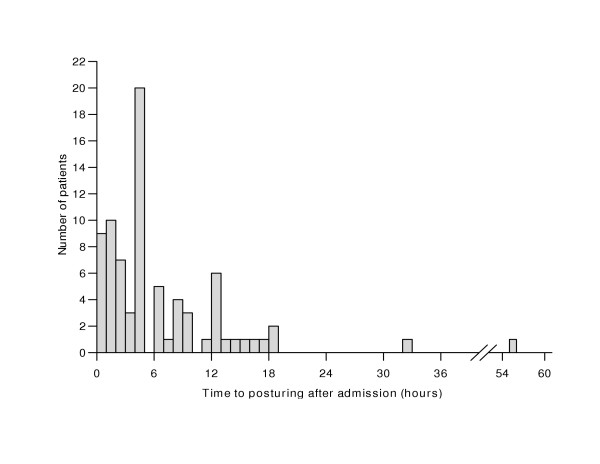
Time from admission to onset of posturing.

The median time from admission to the time of AMP was 4.0 (IQR 1.9 – 8.8) hours. Fifty-two patients developed posturing within six hours and the remaining 26 patients had posturing after six hours (Figure 1). Two children developed opisthotonus 32 and 55 hours after admission. No significant age, sex or clinical differences were observed between those who developed posturing before or after six hours. However, most of those who developed opisthotonic posturing after admission did so after more than six hours of admission: of the 26 patients who developed AMP after 6 hours, 6 (23.3%) had opisthotonic posturing compared to only four out of 52 (7.7%) with AMP within six hours of admission, (OR 2.0 95% CI 1.1 – 3.8). Features of RICP on funduscopy (congested retinal veins, blurred disc margins or overt papilloedema) were the only clinical feature predictive for the development of posturing after admission, (adjusted OR 1.9 95% CI 1.7 – 2.2, *p *< 0.001).

### Clinical events after admission

Deterioration in consciousness observed during the four hourly assessments, recurrence of seizures and brainstem features of raised ICP were the most significant clinical events after admission. The level of consciousness deteriorated in over 50% of patients with AMP, within 24 hours of admission and often, just before further posturing episodes (Table 1).

### Abnormal motor posturing and features of intracranial hypertension

Several brainstem signs consistent with different stages of raised ICP were observed. Abnormalities of pupillary size and reaction, eye movements, disorders of conjugate gaze, and changes on funduscopy were the most common features (Table 1). Motor abnormalities of tone and reflexes were also seen. Raised ICP was associated with all three types of posturing (Table 2), although patients with decorticate posturing had earlier funduscopic features such as congested retinal veins, unlike those with decerebrate posturing who had papilloedema more frequently.

**Table 2:** Brainstem features of raised ICP, seizures after admission and type of posturing

**Brainstem signs and seizures after admission**	**No Posturing (%)**	**Decorticate posturing (%)**	**P value**	**Decerebrate posturing (%)**	**P value**	**Opisthotonus posturing (%)**	**P value**
Number	254	80		61		22	
Deterioration in level of consciousness	64 (25.2)	43 (53.8)	< 0.001	25 (41.0)	0.01	14 (63.6)	<0.001
Pupillary reaction							
Sluggish	82 (32.4)	32 (40.0)	0.21	25 (41.0)	0.20	10 (45.5)	0.21
Fixed	12 (4.7)	1 (1.3)	0.32*	4 (6.6)	0.52*	2 (9.1)	0.31*
Pupil sizes							
Constricted	42 (16.5)	6 (7.5)	0.05	5 (8.2)	0.10	3 (13.6)	1.00*
Dilated	41 (16.2)	20 (25.0)	0.08	17 (27.9)	0.03	6 (27.3)	0.19
Unequal	3 (1.2)	2 (2.5)	0.60*	3 (4.9)	0.09*	1 (4.5)	0.28*
Dysconjugate vision	42 (17.1)	27 (33.8)	< 0.01	13 (21.3)	0.44	8 (36.3)	0.02
Ocular fundi							
Congested retinal veins	11 (4.3)	11 (13.8)	< 0.01	2 (3.3)	1.00*	4 (18.2)	0.02*
Blurred disc margins	6 (2.4)	6 (7.5)	0.03	6 (9.8)	<0.01	2 (9.1)	0.13*
Papilloedema	4 (2.4)	3 (3.8)	0.37*	7 (11.5)	<0.001	1 (4.5)	0.34*
Retinal haemorrhages	18 (7.1)	12 (15)	0.03	11 (18.0)	<0.01	3 (13.6)	0.23*
Any fundoscopic evidence of Raised Intracranial Pressure	16 (6.3)	15 (18.8)	< 0.01	12 (19.7)	0.001	5 (22.7)	<0.01
Horizontal oculocephalic deviation							
No deviation	17 (6.7)	6 (7.5)	0.80	3 (4.9)	0.78*	2 (9.1)	0.66*
Minimal deviation	44 (17.3)	12 (15)	0.63	10 (16.4)	0.86	5 (22.7)	0.53
Respiration							
Irregular	49 (19.3)	13 (16.3)	0.54	20 (32.8)	0.02	5 (22.7)	0.70
Shallow	42 (16.5)	5 (6.3)	0.02	12 (19.7)	0.56	3 (13.6)	1.00*
Respiratory arrest with good cardiac Output	12 (4.7)	10 (12.5)	0.01	6 (9.8)	0.12	3 (13.6)	0.11*
Clinical seizures observed in the ward	129 (50.8)	48 (60.0)	0.15	42 (68.9)	0.01	17 (77.3)	0.02
5 or more seizures in the ward	29 (11.4)	13 (16.3)	0.26	13 (21.3)	0.04	6 (27.3)	0.03
Type of clinical seizure							
Partial	74 (29.1)	31 (38.8)	0.11	22 (36.1)	0.29	13 (59.1)	<0.01
Partial with secondary generalization	29 (11.4)	13 (16.3)	0.26	9 (14.8)	0.47	5 (22.7)	0.12
Generalised	81 (31.9)	29 (36.3)	0.47	30 (49.2)	0.01	8 (36.4)	0.67

* Fisher's exact test

Thirty-two patients thought to have severe intracranial hypertension usually with papilloedema or clinical signs compatible with progressive herniation (23 of whom had AMP), were given mannitol (0.5 g/kg infused over 20 minutes up to a maximum of three doses). No further episodes of AMP were observed in 12 (52.2%) after the administration of mannitol. Fifteen of the 32 patients (46.9%) died and five (15.6%) showed severe neurological deficits. Fourteen of the 15 deaths occurred in patients with AMP.

### Herniation syndromes

Patients were assessed for features of transtentorial herniation based upon the Plum and Posner criteria adapted for children with cerebral malaria [5,11]. Forty-eight patients (11.5%) fulfilled a criterion for herniation (Table 3). Thirty three (68.8%) of these died compared to only 30 (8.1%) of those without features of herniation, (OR 12.4 95% CI 7.1 – 21.4, *p *< 0.001). Most patients with AMP who died had features of herniation (19/31), with most deaths following a respiratory arrest.

**Table 3:** Herniation syndromes and posturing

**Herniation syndromes**	**Posturing (n = 163)**	**No posturing (n = 254)**	**P Value**
Uncal^1^	2 (1.2)	2 (0.8)	0.64
Diencephalic^2^	8 (4.9)	2 (0.8)	0.02
Midbrain/upper pontine^3^	0 (0.0)	2 (0.8)	0.52
Lower pontine^4^	2 (1.2)	1 (0.4)	0.56
Medullary^5^	19 (11.7)	12 (4.7)	0.01

^1 ^Unilateral mydriasis and unilateral fixed pupil, unilateral ptosis, minimal or no oculocephalic eye deviation and hemiparesis

^2 ^Cheynes-Stokes respiration, small or midpoint pupils reactive to light, full deviation of oculocephalic response, flexor response to pain and/or decorticate posturing, hypertonia and or hypereflexia with extensor planters

^3 ^Hyperventilation, midpoint pupils fixed to light, minimal oculocephalic deviation, extensor response to pain or decerebrate posturing

^4 ^Shallow or ataxic respiration, midpoint pupils fixed to light, no oculocephalic response, flexion of legs only, no response to pain or decorticate posturing, flaccid with extensor planters

^5 ^Slow irregular or gasping respirations, dilated pupils fixed to light, respiratory arrest with adequate cardiac output

### Seizures after admission

Clinical seizures were observed in 107/163 (65.6%) patients with AMP compared to 129/254 (50.8%) of those without AMP (Table 1). Most patients with AMP had multiple short seizures, often lasting one to three minutes and 20% had five or more seizures witnessed over the duration of admission. Seizures were particularly common among those who developed AMP after admission, often involving the mouth, face and limbs and were twice as common as that in patients without AMP. An association was observed between the type of AMP and recurrence of seizures in the ward; no posturing 50.8%, decorticate posturing, 60.0%, decerebrate, 68.9% and opisthotonus, 77.3% (χ^2 ^= 11.4, *p *= 0.01 for trend). The median number of witnessed seizures among patients with opisthotonus was three compared to one in patients with either decerebrate or decorticate posturing.

Thirty five of the 85 patients with AMP at admission received intravenous phenytoin. AMP recurred in 22/35 (62.9%) of those who received phenytoin compared to 41/50 (82.0%) of those who did not receive phenytoin, but this difference was not statistically significant.

### Abnormalities on cerebral function analyser monitoring

**Figure 2:**
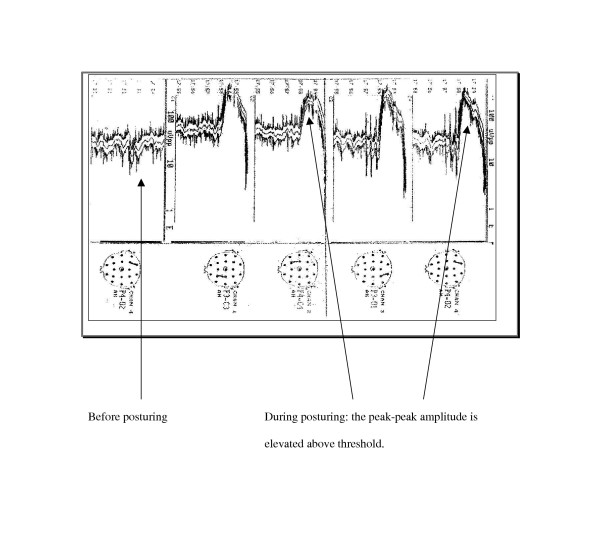
Cerebral function analyser monitoring tracings before and during opisthotonic posturing in a child with cerebral malaria.

Twenty-one of the 58 patients (36%) monitored with the CFAM had AMP, a proportion similar to that seen in whole group. Recordings were examined for evidence of seizure activity and changes in background electrical activity during AMP. Patients with AMP had elevated peaks in the amplitude of the background electrical activity, which were not seen in those without AMP. During posturing, transient increases in the amplitude of the electroencephalographic (EEG) background activity were observed (Figure 2). These were most marked with opisthotonic posturing and more pronounced with decerebrate than with decorticate posturing. The mean peak-to-peak amplitude in patients with decorticate posturing increased to just below 100 μV (CFAM voltage readings are in a logarithmic scale starting at 1, 10 and 100 μV), while in decerebrate and opisthotonic posturing it exceeded 100 μV. There was no evidence of seizure activity during AMP in the areas monitored by EEG (left and right fronto-central: F3-C3, F4-C4; left and right parieto-occipital: P3-O1, P4-O2).

### Seizures, posturing and resolution of coma

Both seizures and AMP were associated with prolonged coma. The median time to full consciousness in patients with AMP was 32 (IQR 12–63) compared to 13 (IQR 4–26) hours in those without AMP. In patients who had seizures after admission, the median time to full consciousness was 26 (IQR 10.3–51.8) hours compared to nine (IQR 4–21.5) among those without seizures. On multiple linear regression analysis, time to full consciousness was significantly longer in both patients with AMP, (β = -21.4, t = -5.453, *p *< 0.001) and those with seizures after admission, (β = -20.9, t = -5.485, *p *< 0.001). Of the 374 patients alive 24 hours after admission, 163/232 (70.3%) without AMP had regained full consciousness compared to only 57/142 (40.1%) with AMP, *p *< 0.001. Prolonged periods of unconsciousness were particularly common in patients who developed AMP after admission when compared with those posturing at admission, 53.8 (SD 55.1) vs 30.8 (SD 33.8) hours, *p *= 0.002.

### Independent risk factors for abnormal motor posturing

Logistic regression analysis was performed to determine the main risk factor for AMP in particular, features associated with raised ICP or seizures. Only features of raised ICP on funduscopy (adjusted OR 2.1 95% 1.2 – 3.7, *p *= 0.009) were independently associated with AMP. No independent association was observed with seizures. When the different types of AMP were considered separately, decorticate posturing was associated with raised ICP (adjusted OR 3.2 95% CI 1.5–6.9, *p *= 0.003) but not seizures (adjusted OR 1.3 95% CI 0.7–2.1, *p *= 0.378). However, decerebrate posturing was significantly associated with both raised ICP (adjusted OR 3.2 95% CI 1.4–7.1, *p *= 0.006) and seizures (adjusted OR 1.9 95% CI 1.0–3.5, *p *= 0.036). Similarly, opisthotonic posturing was also associated with both raised ICP (adjusted OR 3.4 95% CI 1.1–10.7, *p *= 0.035) and seizures (adjusted OR 2.9 95% CI 1.0–8.1, *p *= 0.049).

### Outcome

Out of the 417 children with cerebral malaria, 63 (15.1%) died and 39 (11.0%) of the 354 survivors had neurological deficits at discharge. The mortality in the 163 patients with AMP was 19.0% compared to 12.6% of 254 patients without AMP, *p *= 0.07. Primary respiratory arrest was the commonest cause of death among patients with AMP (19/31 [61.3%] compared to 12/32 [37.5%] in those without posturing, *p *= 0.06) and the median time from admission to death was four hours longer (15 hours IQR 3–48 vs 11.5 hours IQR 3–106) among patients with AMP. Mortality increased with the type of AMP: mortality was 12.6% among children without AMP, 16.3% in those with decorticate posturing, 19.7% with decerebrate posturing and 27.3% in those with opisthotonic posturing. Patients who developed AMP after admission had the worst outcome; 40% either died or had gross neurological deficits at discharge (Table 1).

At discharge, of the 39 children with neurological deficits, motor impairments in 28 (18 with central hypotonia, six with paresis and four with ataxia), aphasia in 13, blindness in 12 and deafness in 6 were the most common impairments. These impairments were particularly common among patients with AMP: 22 (16.7%) compared to 17 (7.7%) of those without AMP, *p *= 0.009). Other neurological deficits observed in patients with AMP included labile emotions with frequent mood changes, persistent visual hallucinations, lip smacking, choreoathetosis and repeated episodes of confusion. Recurrence of seizures in the ward was also associated with both increased neurological deficits (16.6 vs 4.3%, OR 4.4 95% CI 1.9–10.2, *p *< 0.001) and mortality (18.2 vs 11.0%, OR 1.8 95% CI 1.0–3.2, *p *= 0.04).

## Discussion

This study has shown that abnormal motor posturing is a common feature of cerebral malaria in Kenyan children, occurring in 40% of patients. It is associated with features of raised ICP, seizures after admission, prolonged coma, increased mortality and neurological deficits. The evidence suggests that raised ICP may be the most important risk factor.

### Risk factors for posturing

With the exception of age, patients with or without AMP had similar demographic and clinical characteristics. The reduced risk of posturing in younger children may be explained by an open fontanel or uncalcified sutures that can dissipate increases in intracranial pressure. There was no association with well recognised metabolic causes such as hypoglycaemia or hyponatraemia [10,11]. Evidence of raised ICP on funduscopy was the only risk factor that predicted AMP after admission. Decorticate posturing was associated with features of raised ICP only while decerebrate and opisthotonic posturing were associated with features of raised ICP and seizures after admission. Opisthotonic posturing had a third independent risk factor, severe metabolic acidosis. It would appear that although features of raised ICP are strongly associated with AMP, the type of AMP may be influenced by the degree of raised ICP and the presence of seizures or acidosis. Decorticate posturing developed in patients with earlier features of raised ICP such as congested retinal veins while decerebrate posturing was associated with a more overt feature of raised ICP, papilloedema, and with recurrent seizures. Opisthotonic posturing was independently associated with features of raised ICP, recurrent seizures and severe metabolic acidosis.

The clinical significance of the risk factors associated with the different types of AMP is not clear. Sherrington originally described decorticate and decerebrate AMP in the context of the trans-section of the brain of a cat at different levels[21]. Since then, AMP has been incorporated in the clinical assessment of coma. Raised ICP and coning are presumed to cause compression of the brainstem resulting in an ischaemic trans-section of the cerebrum, "separating" it from the brainstem [11,22,23]. This possibly may be the predominant mechanism in the causation of AMP in children with cerebral malaria, given the considerable clinical evidence for raised ICP observed in these patients.

### Abnormal motor posturing, seizures and raised ICP

AMP was associated with recurrence of seizures after admission, mostly as short multiple seizures, but not seizures before admission. CFAM recordings showed increases in the mean peak-to-peak amplitude of the EEG background activity, most marked during opisthotonic posturing. These amplitude increases may possibly reflect acute elevations of ICP. There was no indication of any epileptiform activity during AMP recorded from the four leads of the CFAM. It is however possible that these leads were not able to detect localized discharges.

The association between the more severe forms of AMP – decerebrate and opisthotonic posturing – and seizures may arise because seizures increase ICP, PCO_2 _and cerebral blood flow, which then leads to AMP. Increased metabolic demands and cerebral blood flow during seizures may aggravate the critical perfusion state. It is less plausible that AMP itself represents an ictal phenomenon, since there is no evidence of a temporal association between seizure activity on CFAM and the occurrence of posturing. Continuous ICP and EEG monitoring would allow further investigation of the relationship between electrical seizures and AMP.

### Posturing and outcome

In many encephalopathies, AMP is associated with a poor prognosis [3,10]. A similar trend was observed in this study. In addition to being a sign of lower brainstem compression, the particularly high mortality in patients with opisthotonic posturing may be a result of the combined effects of a more severe encephalopathy with raised ICP, repeated seizures and severe metabolic acidosis. Severe metabolic acidosis in African children with malaria has been associated with shock/hypovolaemia, anaemia, lactic acidosis and other ketones [24,25]. Those with cerebral malaria and concurrent severe metabolic acidosis have the highest severe malaria mortality [26]. The association of opisthotonic posturing with severe acidosis suggests that this type of abnormal posturing develops in very sick patients with the highest risk of death.

The median time to death in patients with AMP was four hours longer than that in patients without AMP and mortality was highest in those who developed AMP after six hours of admission. The majority of deaths had a respiratory arrest with initially an adequate cardiac output, a phenomenon associated with medullary herniation syndrome [5,7,11]. Herniation syndromes could be recognized in two-thirds of deaths in patients with posturing and over 50% of all deaths [13]. Effective treatment of intracranial hypertension may, therefore, be able to reduce the mortality of cerebral malaria in African children especially in patients who develop posturing after admission. The additional four hours in the median time to death provide an opportunity for the use of measures aimed at lowering ICP. Patients that develop AMP after admission may be the best group to target.

### Posturing and therapy for raised ICP

No guidelines exist for the use of mannitol in cerebral malaria [2], but it was administered to 32 patients in this series. Despite mannitol administration, 15 patients died and another five developed severe neurological deficits. Uncontrolled studies of mannitol in cerebral malaria show that it reduces ICP but may not prevent subsequent development of severe intracranial hypertension [5]. The use of mannitol or other osmotic diuretics requires further investigation. Most district hospitals across sub-Saharan Africa that also treat the majority of patients with cerebral malaria are unable to offer ventilation to patients with raised ICP [27]. Since most patients develop AMP several hours before death, posturing may be used as an early marker for clinicians to start initial treatment for raised ICP: patient positioning, adequate oxygenation and perfusion, temperature and seizure control, and possibly offer mannitol therapy. This can be done with the hope that at this stage, patients still have mild or moderately elevated intracranial pressures that may respond to mannitol [7]. However, the use of osmotic diuretics may have risks that can ideally be minimised by more intensive monitoring including that of ICP.

## Conclusion

In conclusion, AMP is a common feature of cerebral malaria especially in older children. Such patients are at an increased risk of multiple seizures after admission, prolonged coma, increased mortality and neurological deficits. Although raised ICP appears to be the most important risk factor for AMP, the association between the more severe forms of AMP and seizures needs to be further investigated. Continuous multi-lead EEG and ICP monitoring, a randomized trial of seizure prophylaxis and/or treatment for raised ICP may be useful.

## Authors' contributions

RI designed the study, analysed the data and wrote the first draft. GO and BO carried out clinical care for the patients, performed the CFAM recordings and participated in data interpretation. SW analysed the CFAM recordings, participated in data analysis and interpretation. AK carried out clinical care of the patients. GF, SM, KM and BGRN participated in data analysis and interpretation. CRJCN participated in study design, data analysis and interpretation. All authors critically reviewed the manuscript.

## Supplementary Material

Additional File 1 Demographic and clinical features of children with cerebral malaria with or without abnormal motor posturing. Click here for file
