# Supplementary material for: Decorticate, decerebrate and opisthotonic posturing and seizures in Kenyan children with cerebral malaria
Source: Malar J. 2005 Dec 7;4:57. doi: 10.1186/1475-2875-4-57 (PMC1326205; doi:10.1186/1475-2875-4-57)
Supplement: Additional File 1 — Demographic and clinical features of children with cerebral malaria with or without abnormal motor posturing. [file 1475-2875-4-57-S1.doc]

**Supplemental file**

**Demographic and clinical features of children with cerebral malaria with or without abnormal motor posturing**

| **Demographic and clinical features** | **Patients with AMP**  **(n = 163)** | **Patients without AMP**  **(n = 254)** | ***P***  **value** |
| --- | --- | --- | --- |
| Sex, male (%) | 52.1 | 51.2 | 0.920 |
| Mean age (SD) in months | 35.5 (23.5) | 28.3 (20.1) | 0.001 |
| Median (IQR) duration of illness in days | 2 (2 – 3) | 2 (1 – 3) | - |
| Anti malarial before admission, (%) | 46.6 | 38.9 | 0.149 |
| Median (IQR) duration of coma before admission, (hrs) | 3 (2 – 6) | 3 (2 – 6) | - |
| History of convulsions, (%) | 85.2 | 85.4 | 1.000 |
| Mean axillary (SD) temperature, ^o^C | 38.7 (7.0) | 38.0 (4.3) | 0.162 |
| Depth of coma, (%)  Blantyre Coma Score = 0  Blantyre Coma Score = 1  Blantyre Coma Score = 2 | 20.9  28.2  50.9 | 22.8  26.4  50.8 | 0.861 |
| Geometric mean parasite density/μl | 55611 | 60035 | - |
| Mean (SD) haemoglobin, (g/dl) | 7.4 (2.4) | 6.9 (2.4) | 0.059 |
| Mean (SD) serum sodium, (mmol/L) | 135.0 (5.7) | 134.4 (5.0) | 0.278 |
| Multiple convulsions[[1]](#footnote-2), (%) | 40.5 | 44.9 | 0.418 |
| Prolonged coma[[2]](#footnote-3), (%) | 10.0 | 9.3 | 0.812 |
| Deep (acidotic) breathing, (%) | 30.7 | 27.2 | 0.439 |
| Hypotension[[3]](#footnote-4), (%) | 14.8 | 16.9 | 0.579 |
| Hypoglycaemia[[4]](#footnote-5), (%) | 20.4 | 21.3 | 0.899 |
| Severe anaemia[[5]](#footnote-6), (%) | 19.5 | 22.3 | 0.534 |
| Hyperparasitaemia[[6]](#footnote-7), (%) | 28.9 | 31.8 | 0.581 |
| Severe acidosis[[7]](#footnote-8), (%) | 29.0 | 24.6 | 0.398 |
| Hyponatraemia[[8]](#footnote-9), (%) | 47.7 | 47.7 | 1.000 |
| Deterioration in level of consciousness within 24 hrs of admission, (%) | 50.3 | 25.2 | <0.0001 |
| Clinical seizures observed in the ward, (%) | 65.6 | 50.8 | 0.003 |
| Type of clinical seizure, (%)  Partial  Partial with secondary generalisation  Generalised | 40.5  16.6  41.1 | 29.1  11.4  31.9 | 0.017  0.133  0.055 |
| Status epilepticus in the ward, (%) | 26.4 | 20.9 | 0.193 |

1. Three or more convulsions before admission [↑](#footnote-ref-2)
2. Coma >12 hours before admission [↑](#footnote-ref-3)
3. Mean arterial blood pressure < 60 mmHg [↑](#footnote-ref-4)
4. Blood sugar <2.2 mmol/L [↑](#footnote-ref-5)
5. Haemoglobin <5.0 g/dl [↑](#footnote-ref-6)
6. Parasite density >500,000/μl [↑](#footnote-ref-7)
7. Base excess < -15 [↑](#footnote-ref-8)
8. Serum sodium <135 mmol/L [↑](#footnote-ref-9)
